# Context-dependent aesthetic perception after facial local flap reconstruction for cutaneous malignancies: a multi-observer study integrating patient-reported outcomes and public perspective

**DOI:** 10.3389/fsurg.2026.1982895

**Published:** 2026-10-05

**Authors:** Ersin Yavuz, Esra Zeynep Kurtoğlu, Levent Demir, Alp Ercan

**Affiliations:** 1 Department of Plastic, Reconstructive and Aesthetic Surgery, Diyarbakır Gazi Yaşargil Training and Research Hospital, Diyarbakır, Türkiye; 2 Department of Plastic, Reconstructive and Aesthetic Surgery, Istanbul University-Cerrahpasa, Istanbul, Türkiye

**Keywords:** aesthetic perception, cutaneous malignancy, facial reconstruction, local flaps, patient-reported outcomes

## Abstract

**Background:**

Local flaps are fundamental for reconstruction of facial defects after cutaneous tumor resection, yet perceived aesthetic success may differ among surgeons, patients, and the general public.

**Objectives:**

To investigate context-dependent aesthetic perception across facial regions and evaluator groups and its relationship with patient-reported FACE-Q outcomes.

**Methods:**

Twenty-three patients undergoing local flap reconstruction after facial tumor resection were evaluated using preoperative and 12-month postoperative photographs. Overall, 203 evaluators—plastic surgeons, non-plastic surgeons, non-surgical physicians, and the general public—rated outcomes on a five-point scale, first using postoperative images alone and subsequently after viewing corresponding preoperative photographs. FACE-Q Face Overall, Natural Appearance, and Psychological Function scales were assessed preoperatively and postoperatively.

**Results:**

Preoperative context resulted in significantly more critical ratings across perioral, periocular, and malar regions (all *p*<0.001). Perceptual shifts differed among evaluator groups in perioral (*p*=0.007) and periocular (*p*=0.015) regions, with plastic surgeons showing smaller shifts. FACE-Q Face Overall and Natural Appearance scores improved significantly postoperatively. A study-specific summary score derived from the three selected FACE-Q scales was significantly associated with general-public ratings (*ρ* = −0.56, *p* = 0.008).

**Conclusions:**

Aesthetic assessment after facial local flap reconstruction is context-dependent. Integrating FACE-Q patient-reported outcomes with public perception may provide a more patient-centered assessment of reconstructive success.

## 1 Introduction

Facial reconstruction represents a major challenge in reconstructive surgery due to the face's complex three-dimensional anatomy, its central role in communication and expression, and its importance for identity and social interaction (1, 2). Achieving optimal outcomes requires not only restoration of function but also aesthetically harmonious results, necessitating careful integration of anatomical, biomechanical, and aesthetic principles.

The concept of facial aesthetic units is widely adopted to improve reconstructive outcomes by restoring natural contours, minimizing visible scarring, and preserving facial harmony. Initially described by González-Ulloa and later refined by Burget and Menick, particularly in nasal reconstruction, this concept divides the face into regions with similar skin characteristics such as color, texture, thickness, and mobility (3–5). These units—including the forehead, periocular region, nose, lips, cheek, and chin—allow reconstruction to follow natural boundaries, thereby improving aesthetic integration compared with defect-based repair alone.

Despite extensive descriptions of aesthetic unit–based reconstruction in surgical planning and structural outcomes, limited attention is given to how these region-specific approaches are perceived by different observers. In clinical practice, postoperative appearance is inherently subjective and influenced by surgical expertise, medical background, and lay perception. Patient expectations further shape satisfaction, while differences between specialist and non-specialist observers may influence communication and clinical decision-making.

Aesthetic evaluation is also influenced by contextual factors. Perceived outcomes may change when the appearance and extent of the preoperative lesion are considered, particularly in visually sensitive facial subunits (6). However, this contextual effect is not systematically evaluated across facial regions or observer groups in local flap reconstruction.

Surgeons typically assess outcomes based on technical success and adherence to reconstructive principles; however, these criteria may not fully reflect how reconstructed faces are perceived by patients or the general public. Therefore, discrepancies between surgical evaluation and real-world perception may exist.

Accordingly, this study aims to evaluate the aesthetic outcomes of facial local flap reconstruction across different facial aesthetic subunits using a structured perception-based approach. Specifically, it examines whether the effect of preoperative contextual information on postoperative aesthetic assessment differs according to evaluator background and facial region, whether evaluator-related differences in contextual reassessment vary across facial regions, and explores the relationship between patient-reported outcomes (FACE-Q) and observer-based aesthetic evaluations.

## 2 Materials and methods

Following approval by the institutional review board (Approval No: 1ikK1m55), written informed consent was obtained from all patients. This study was designed as a retrospective observational analysis of patients who underwent reconstructive procedures for post-oncologic facial defects.

An initial cohort of 50 consecutive patients who underwent facial reconstruction using local flap techniques following tumor resection between January 2024 and December 2025 was retrospectively reviewed. Patients were excluded if they declined participation in the study, had a history of multiple previous local flap reconstructions in the same facial region, or lacked complete preoperative or 12-month postoperative photographic documentation. Of the 50 patients assessed for eligibility, 27 were excluded: 14 declined participation, 8 had undergone multiple previous local flap reconstructions in the same facial region, and 5 lacked complete preoperative or 12-month postoperative photographic documentation. The remaining 23 patients were included in the final analysis. Of the included patients, 13 were male and 10 were female**.** Patient age ranged from 38 to 72 years**,** with a mean age of 43 years**.** The underlying oncologic diagnoses consisted of basal cell carcinoma in 14 patients**,** squamous cell carcinoma in 7 patients and malignant melanoma in 2 patients**.**

Local flap selection was performed based on defect location, size, involvement of adjacent aesthetic subunits, and functional requirements**.** In the perioral region, reconstruction was achieved using a bilateral Karapandzic flap in 7 patients and an Estlander flap in 6 patients**,** depending on defect extent and commissure involvement. For periocular and malar reconstructions, a variety of region-appropriate local flaps were utilized. Nasolabial V–Y advancement flaps were performed in 3 patients**,** glabellar flaps in 4 patients, and Mustarde cheek rotation flaps in 3 patients**,** selected according to defect geometry and the need to preserve eyelid position and overall facial symmetry. All reconstructive procedures were designed to respect facial aesthetic unit boundaries and to optimize both functional preservation and aesthetic integration.

Clinical photographs were obtained preoperatively and at 12 months postoperatively using a smartphone camera at 1.8× magnification. Because this was a retrospective study, image acquisition was not performed according to a prospectively predefined photographic protocol with fixed camera-to-subject distance, identical lighting conditions, uniform background, or strictly standardized head position and facial expression. For the perception-based assessment, photographs were selected to provide the clearest available representation of the relevant facial region and reconstructive outcome. Images were digitally displayed to all evaluators on a computer screen in a consistent presentation format, with the relevant facial region clearly visible. Additional image magnification or zooming was not permitted during the assessment. No geometric normalization or three-dimensional registration was performed.

Aesthetic outcomes were evaluated by an independent assessment panel comprising four distinct evaluator groups: board-certified plastic surgeons (*n* = 53), non-plastic surgeons (*n* = 50), non-surgical physicians (*n* = 57), and members of the general public (*n* = 43), yielding a total of 203 evaluators. Evaluators ranged in age from 35 to 65 years; 103 were male and 100 were female. Evaluators were recruited by direct invitation to physicians and members of the general public. The plastic surgeon group comprised specialists with 2–20 years of post-specialization clinical experience. The non-plastic surgeon group consisted of otorhinolaryngologists and ophthalmologists, representing surgical specialties with clinical involvement in the facial region, whereas the non-surgical physician group consisted of specialists in internal medicine and pediatrics. The general-public group included individuals from heterogeneous occupational and educational backgrounds, including homemakers and professionals such as engineers; no specific medical or surgical expertise was required for inclusion in this group. Previous individual experience with facial reconstruction or oncologic surgery was not separately recorded beyond professional specialty and level of experience. The inclusion of evaluators from different professional backgrounds was intended to capture a broad spectrum of aesthetic perceptions across evaluator groups. All evaluators were blinded to patient identity, clinical history, and surgical details.

Aesthetic evaluation was performed using a standardized two-step photographic assessment protocol**.** In the first step, evaluators reviewed postoperative photographs only and rated aesthetic outcomes using a five-point ordinal scale**,** where lower scores indicated more favorable aesthetic outcomes (Table 1). In the second step, preoperative photographs of the same patients were presented alongside postoperative images, and evaluators reassessed their aesthetic perception using the identical five-point ordinal scale with unchanged response categories and numerical anchors. The two assessments were performed consecutively within the same evaluation session, without a washout interval. The assessment sequence was fixed for all evaluators, with the postoperative-only assessment always preceding the contextual assessment; the order was not randomized or counterbalanced. This two-step approach was designed to examine whether the availability of preoperative contextual information was associated with a change in the aesthetic evaluation of the postoperative outcome.

**Table 1:** Photographic aesthetic assessment protocol.

Evaluation phase	Images presented	Response options
Step 1	Postoperative photographs only	**1-**Indistinguishable from normal anatomy **2-** Close to normal anatomy **3-** Acceptable appearance **4-** Presence of deformity **5-** Presence of severe deformity
Step 2	Postoperative + preoperative photographs	**1-** Indistinguishable from normal anatomy **2-** Close to normal anatomy **3-** Acceptable appearance **4-** Presence of deformity **5-** Presence of severe deformity

All evaluations were performed using the same five-point ordinal aesthetic scale, with lower scores indicating more favorable aesthetic outcomes. In Step 1, postoperative photographs were assessed in isolation. In Step 2, the same postoperative outcomes were reassessed using the identical five-point scale after presentation of the corresponding preoperative photographs. Thus, the response categories and their numerical anchors remained unchanged between the two assessment phases.

In addition to observer-based aesthetic assessment, patient-reported outcomes were evaluated using the FACE-Q | Aesthetics. The FACE-Q is a validated patient-reported outcome measure designed to assess appearance and health-related quality of life following facial aesthetic and reconstructive procedures (7–10). Patients completed three independently functioning FACE-Q scales: Face Overall (Appearance domain), Natural Appearance (Natural domain), and Psychological Function (Health-Related Quality of Life domain), both preoperatively and at the 12-month postoperative follow-up.

Raw summed scores were calculated separately for each FACE-Q scale, with higher scores indicating better patient-reported outcomes.

To explore the relationship between patient-reported outcomes and external aesthetic evaluation, a study-specific summary score was calculated for each patient as the arithmetic mean of the postoperative raw summed scores from the Face Overall, Natural Appearance, and Psychological Function scales. This derived measure was created solely for the exploratory correlation analysis and does not represent a validated FACE-Q scale, domain score, composite score, or overall FACE-Q score. For each patient, median postoperative-only aesthetic scores were calculated within each evaluator group.

The overall methodological framework of the study, including the two-step paired photographic assessment, evaluator stratification, and region-specific analysis, is summarized in Figure 1.

**Figure 1:**
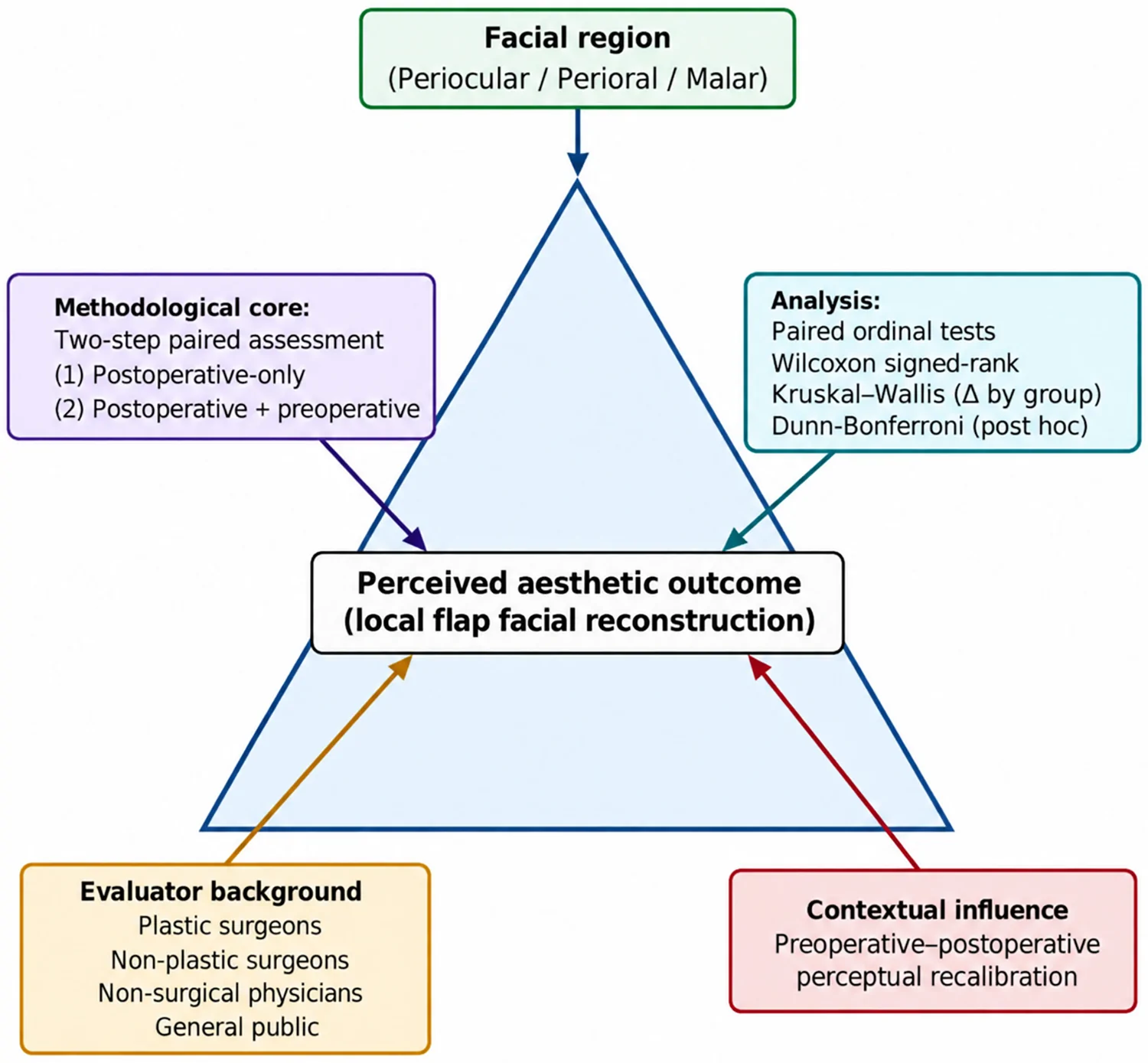
Conceptual framework illustrating the factors influencing perceived aesthetic outcomes after facial local flap reconstruction. A two-step paired photographic assessment protocol was used, consisting of postoperative-only evaluation followed by contextual reassessment incorporating preoperative lesion appearance. Perceived aesthetic outcome represents the central construct and is modulated by facial region (periocular, perioral, and malar),evaluator expertise (plastic surgeons, non-plastic surgeons, non-surgical physicians, and members of the general public) and contextual influence resulting from preoperative–postoperative comparison. The primary inferential analysis used a cross-classified mixed-effects model with evaluator and patient as crossed random effects, while Wilcoxon signed-rank and Kruskal–Wallis/Dunn analyses were used as complementary unadjusted analyses.

### 2.1 Statistical analysis

Statistical analyses were performed using IBM SPSS Statistics for Windows, Version 30.0 (IBM Corp., Armonk, NY, USA). Continuous variables were summarized using means and standard deviations or medians and interquartile ranges, as appropriate, while categorical variables were presented as frequencies and percentages.

Because the aesthetic assessment scores were measured on a five-point ordinal scale and each evaluator assessed multiple patients while each patient was assessed by multiple evaluators, the statistical analyses were structured according to the specific research questions and the cross-classified structure of the data. The primary inferential analysis was a cross-classified mixed-effects model using the individual rating-level data. Evaluator and patient were included as crossed random intercepts to account for repeated observations at both levels. Assessment condition (postoperative-only vs. contextual), facial region, evaluator category, and their interactions were included as fixed effects. The condition-by-evaluator category interaction examined whether the magnitude of contextual reassessment differed according to evaluator background; the condition-by-region interaction examined whether the contextual effect differed across facial regions; and the condition-by-region-by-evaluator category interaction examined whether evaluator-related differences in contextual reassessment varied across facial regions. Likelihood-ratio tests were used to assess these interaction terms.

Complementary non-parametric analyses were performed to provide clinically interpretable, unadjusted summaries of the direction and magnitude of the observed changes. For each evaluator, region-specific median aesthetic scores were calculated separately for the postoperative-only and contextual assessment conditions. Paired comparisons between the two assessment conditions were performed using the Wilcoxon signed-rank test. Differences in change scores among evaluator categories were assessed using the Kruskal–Wallis test, followed by Dunn's *post hoc* test with adjustment for multiple comparisons when appropriate. These non-parametric analyses were considered supportive of the primary mixed-effects analysis and were used to facilitate interpretation of the observed rating patterns rather than as the primary basis for inference regarding evaluator- or region-dependent contextual effects.

Preoperative and 12-month postoperative FACE-Q scores were compared using the Wilcoxon signed-rank test. Mean ± standard deviation values were retained as descriptive measures of the FACE-Q score distributions, while median change was used to describe paired within-patient change. Associations between the postoperative study-specific summary score derived from the three selected FACE-Q scales and the corresponding patient-level median postoperative-only aesthetic rating were examined separately for each evaluator group using Spearman's rank correlation coefficients.

Interobserver agreement was additionally assessed within each evaluator group using Krippendorff's alpha with an ordinal distance metric, which accounts for the ordered nature of the five-point aesthetic rating scale and accommodates multiple raters. Agreement was calculated separately for the postoperative-only and contextual assessment conditions across the 23 evaluated patients.

All statistical tests were two-sided, and a *p* value <0.05 was considered statistically significant.

## 3 Results

Of 50 consecutive patients assessed for eligibility between January 2024 and December 2025, 27 were excluded according to the predefined criteria, leaving 23 patients in the final analytic cohort (Figure 2). A total of 203 evaluators independently assessed postoperative outcomes using clinical photographs. For each evaluator, region-specific median aesthetic scores were calculated across all available cases within the perioral (13 cases), periocular (8 cases), and malar (2 cases) regions. Paired comparisons were then performed between postoperative-only assessments and contextual reassessments following exposure to preoperative photographs.

**Figure 2:**
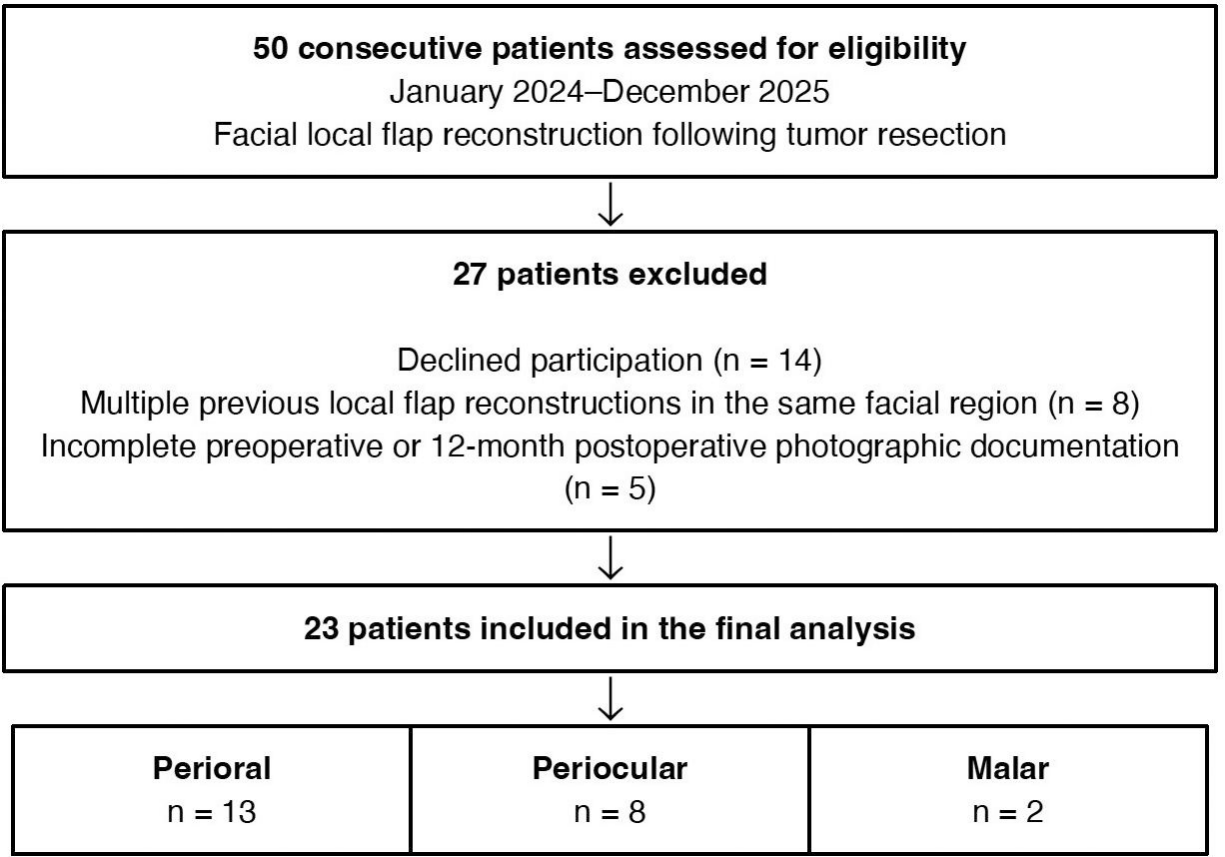
Patient selection flow diagram. Flow diagram illustrating patient identification, exclusion, and final inclusion. Fifty consecutive patients who underwent facial local flap reconstruction following tumor resection between January 2024 and December 2025 were assessed for eligibility. Twenty-seven patients were excluded according to the predefined exclusion criteria, resulting in a final analytic cohort of 23 patients distributed across the perioral (*n* = 13), periocular (*n* = 8), and malar (*n* = 2) regions.

Representative examples of periocular, perioral, and malar local flap reconstructions included in the perception analysis are shown in Figure 3.

**Figure 3:**
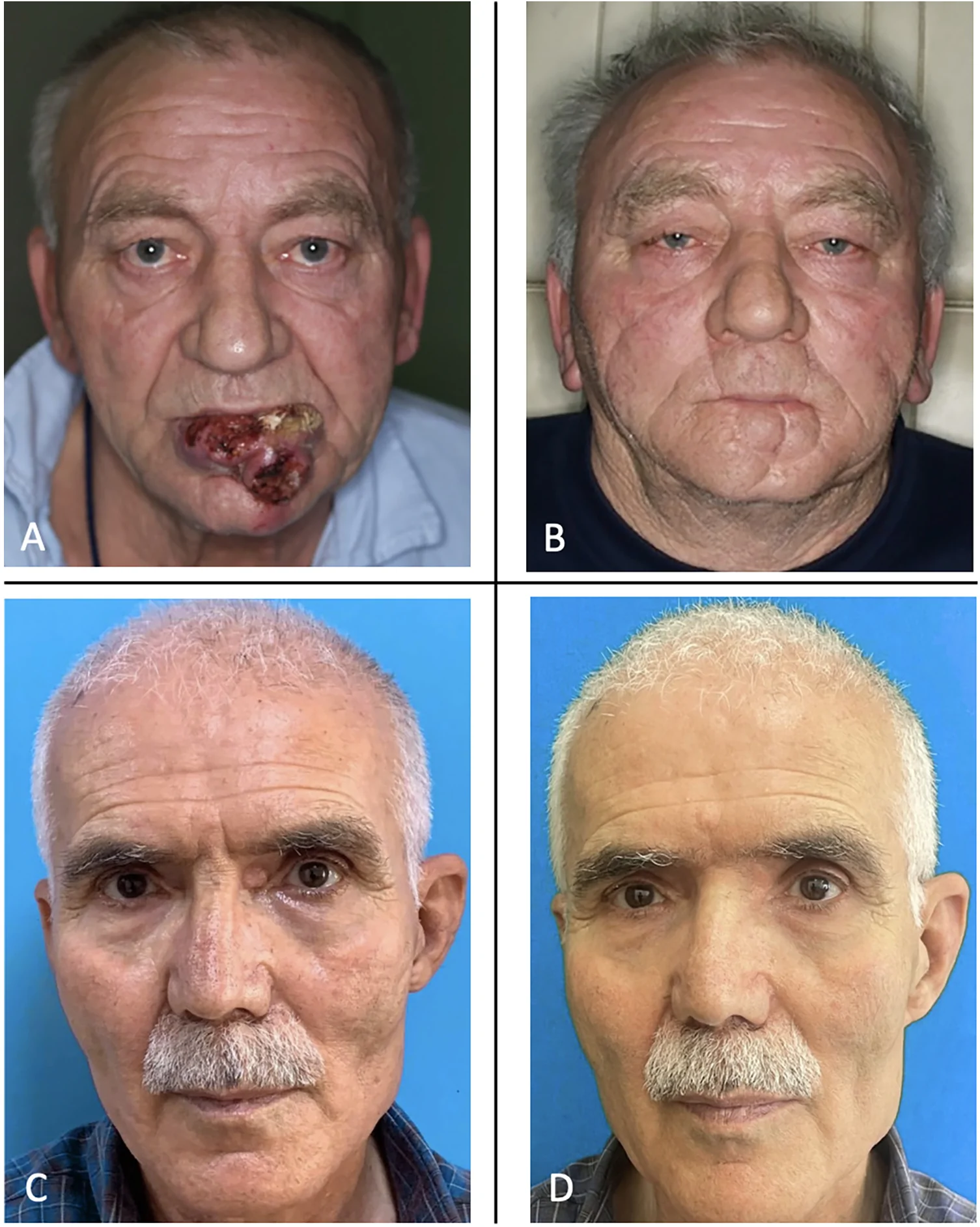
Representative examples of facial local flap reconstructions included in the perception-based analysis. **(A)** Preoperative appearance of a perioral lesion prior to oncologic resection. **(B)** Twelve-month postoperative outcome of the same perioral reconstruction following local flap repair. **(C)** Preoperative appearance of a periocular lesion prior to oncologic resection. **(D)** Twelve-month postoperative outcome of the same periocular reconstruction demonstrating restoration of eyelid contour and regional symmetry.

### 3.1 Region-specific paired analysis

Across all facial regions, paired analysis demonstrated a statistically significant increase in aesthetic scores following contextual evaluation (Wilcoxon signed-rank test, *p* < 0.001 for all regions). In the perioral region, the median postoperative-only score was 2.0, which increased to 5.0 after contextual comparison, yielding a median change (*Δ*) of +2.0 (95% CI: 2.0–3.0). Similarly, periocular reconstructions demonstrated a median postoperative-only score of 2.0 and a contextual median of 5.0, corresponding to a median change of +2.5 (95% CI: 2.0–3.0). In the malar region, the median postoperative-only score was 2.5 and the contextual median was 4.0, with a median change of +2.0 (95% CI: 1.5–2.0). All relevant data are summarized in Table 2.

**Table 2:** Region-level paired effect.

Region	*n* (evaluators)	Median Postop	Median Preop-contextual	Median Δ	95% CI for *Δ*	*p*-value*	Kruskal–Wallis *p* (Δ across evaluator groups)
Perioral (13 cases)	203	2.0	5.0	2.0	2.0 to 3.0	<0.001	0.007*
Periocular (8 cases)	203	2.0	5.0	2.5	2.0 to 3.0	<0.001	0.015*
Malar (2 cases)	203	2.5	4.0	2.0	1.5 to 2.0	<0.001	0.007

Values represent pooled evaluator ratings across cases within each facial region. Median postoperative scores reflect aesthetic assessments based on postoperative photographs alone, whereas median preoperative-contextual scores represent reassessment after exposure to the corresponding preoperative images. Median Δ indicates the paired median change in ordinal aesthetic scores following contextual comparison (contextual score−postoperative-only score), with positive values indicating a shift toward more critical aesthetic evaluation. Ninety-five percent confidence intervals (95% CI) for the median change were estimated using non-parametric methods. Paired comparisons were performed using the Wilcoxon signed-rank test. Differences in the magnitude of perceptual change (Δ) across evaluator groups were assessed using the Kruskal–Wallis test. Values marked with an asterisk indicate statistical significance (*p* < 0.05).

### 3.2 Evaluator-specific regional analysis

When stratified by evaluator background, all evaluator groups demonstrated statistically significant paired changes within each facial region (Wilcoxon signed-rank test, *p* < 0.001 for all comparisons). However, the magnitude of perceptual change varied by both facial region and evaluator expertise. Plastic surgeons consistently exhibited smaller median changes compared with non-plastic surgeons, non-surgical physicians, and members of the general public, particularly in the perioral and periocular regions (Table 3 and Figure 4B). Despite these differences in magnitude, the direction of change was uniform across all evaluator groups, with higher aesthetic scores assigned after contextual evaluation.

**Table 3:** Region and evaluator specific paired changes in aesthetic ratings after contextual exposure.

Facial region	Evaluator group	n	Median post-*p*-only	Median contextual	Median Δ	95% CI for *Δ*	*p*-value
Perioral	Plastic surgeons	53	2.0	4.0	1.0	0.0–3.0	<0.001*
	Non-plastic surgeons	50	2.0	5.0	2.5	2.0–3.0	<0.001*
	Non-surgical physicians	57	2.0	4.0	2.0	2.0–3.0	<0.001*
	General public	43	2.0	5.0	3.0	2.0–3.0	<0.001*
Periocular	Plastic surgeons	53	2.0	4.0	1.0	0.0–2.0	<0.001*
	Non-plastic surgeons	50	2.0	5.0	2.5	2.0–3.0	<0.001*
	Non-surgical physicians	57	2.0	4.0	2.5	2.0–3.0	<0.001*
	General public	43	2.0	5.0	3.0	2.0–3.0	<0.001*
Malar	Plastic surgeons	53	2.5	4.0	0.5	0.0–1.0	<0.001*
	Non-plastic surgeons	50	2.5	4.0	1.5	1.0–2.0	<0.001*
	Non-surgical physicians	57	2.5	4.0	2.0	1.5–2.0	<0.001*
	General public	43	2.5	5.0	2.0	1.5–2.0	<0.001*

This table presents evaluator-stratified paired aesthetic ratings for each facial region. Aesthetic scores are reported as median values for postoperative-only assessment and reassessment after contextual comparison with preoperative images within each evaluator group. Median Δ represents the paired median change in ordinal scores calculated as contextual score−postoperative-only score, with positive values indicating a shift toward more critical aesthetic evaluation. Ninety-five percent confidence intervals (95% CI) for the median change were estimated using non-parametric methods. Values marked with an asterisk indicate statistical significance (*p* < 0.05).

**Figure 4:**
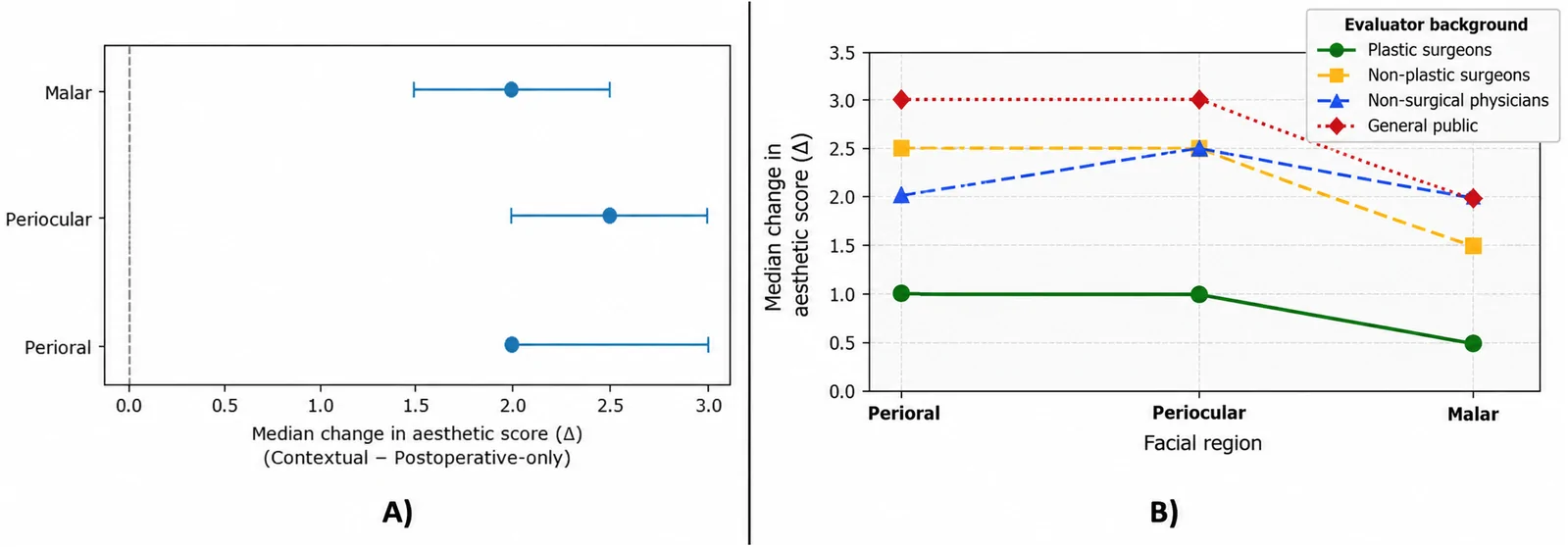
**(A)** Region-specific median change in aesthetic scores following contextual comparison with preoperative photographs. Median change (Δ) was calculated as the difference between preoperative-contextual and postoperative-only assessments. Points represent median differences and horizontal bars indicate 95% confidence intervals. Positive values denote a more critical reassessment after exposure to preoperative images, **(B)** Evaluator-group–specific median changes in aesthetic scores across facial regions. Median changes were calculated as contextual minus postoperative-only scores. Plastic surgeons demonstrated smaller perceptual shifts, whereas non-plastic surgeons, non-surgical physicians, and members of the general public generally showed greater contextual recalibration. Region-specific patterns should be interpreted as exploratory given the limited number of independent patients within individual facial region.

### 3.3 Evaluator-group comparison of perceptual change

To assess whether the degree of perceptual change differed across evaluator groups, median change scores (Δ) were compared using the Kruskal–Wallis test. Significant differences among evaluator groups were observed in the perioral (*p* = 0.007), periocular (*p* = 0.015), and malar (*p* = 0.007) regions (Figure 4B). These unadjusted region-specific findings indicate variation in the magnitude of contextual reassessment across evaluator groups. However, given the limited number of independent patients within individual facial regions, particularly the two malar cases, these findings should be interpreted cautiously and in conjunction with the cross-classified mixed-effects analysis.

### 3.4 Cross-classified mixed-effects analysis

To account for the dependency arising from repeated ratings by the same evaluators and repeated evaluation of the same patients, a cross-classified mixed-effects analysis was additionally performed using 9,310 individual ratings from 203 evaluators and 23 patients. Evaluator and patient were modeled as crossed random effects. In the reference stratum (plastic surgeons evaluating the perioral region), contextual assessment was associated with an increase in aesthetic severity scores compared with postoperative-only assessment (*β* = 0.950, 95% CI 0.836–1.065; *p* < 0.001). Importantly, the magnitude of this contextual change differed significantly across evaluator categories (overall condition-by-evaluator category interaction, *p* < 0.001). In contrast, the condition-by-region interaction did not reach statistical significance (*p* = 0.093), and the three-way interaction between condition, facial region, and evaluator category was also not significant (*p* = 0.631). The estimated random-effect variances were 0.135 at the evaluator level and 0.035 at the patient level. These findings indicate that evaluator background contributed to differences in the magnitude of contextual reassessment, whereas region-specific differences in the contextual effect should be interpreted cautiously.

### 3.5 Interobserver agreement

Ordinal Krippendorff's alpha demonstrated generally low within-group agreement in the postoperative-only assessment, with α values of 0.090 for plastic surgeons, 0.245 for non-plastic surgeons, 0.279 for non-surgical physicians, and 0.368 for members of the general public. Agreement was lower in the contextual assessment, with corresponding α values of 0.008, 0.037, −0.002, and 0.048, respectively. These findings indicate substantial within-group heterogeneity in aesthetic ratings despite the systematic between-group differences observed in the magnitude of contextual reassessment. Accordingly, evaluator-category effects should not be interpreted as implying homogeneous aesthetic judgments within individual evaluator groups.

### 3.6 Patient-reported outcomes

Patient-reported outcomes measured using the FACE-Q demonstrated significant postoperative improvements in two of the three administered scales. The Face Overall scale showed a significant increase from a preoperative mean score of 19.29 ± 2.98 to 28.29 ± 3.59 at the 12-month follow-up (*p* = 0.002). Similarly, the Natural Appearance scale improved significantly, with mean scores increasing from 19.29 ± 2.43 preoperatively to 28.29 ± 3.20 postoperatively (*p* = 0.0021) (Table 4).

**Table 4:** Preoperative and 12-month postoperative FACE-Q scale scores demonstrating patient-reported changes following facial local flap reconstruction.

FACE-Q scale	Preoperative mean ± SD	Postoperative mean ± SD	Median change	*p*-value
Face overall	19.29 ± 2.98	28.29 ± 3.59	+9	0.002*
Natural appearance	19.29 ± 2.43	28.29 ± 3.20	+9	0.0021*
Psychological Function	26.86 ± 3.02	27.86 ± 3.08	+1	0.358

Patient-reported outcomes were assessed using the FACE-Q Face Overall, Natural Appearance, and Psychological Function scales before surgery and at the 12-month postoperative follow-up. Higher scores indicate better patient-reported outcomes. Paired preoperative and postoperative scores were compared using the Wilcoxon signed-rank test. Mean ± standard deviation values are provided descriptively, while median change represents the paired within-patient change. Values marked with an asterisk indicate statistical significance (*p* < 0.05).

In contrast, the Psychological Function scale demonstrated only a modest change, increasing from 26.86 ± 3.02 preoperatively to 27.86 ± 3.08 postoperatively, which did not reach statistical significance (*p* = 0.358). Overall, these findings indicate significant postoperative improvements in Face Overall and Natural Appearance scores, whereas the change in Psychological Function was not statistically significant.

### 3. 7 Relationship between patient-reported satisfaction and observer-based aesthetic perception

Correlation analysis demonstrated varying degrees of association between the postoperative study-specific summary score derived from the three selected FACE-Q scales and observer-based aesthetic evaluations across evaluator groups (Table 5). The summary score showed a correlation of *ρ* = −0.56 (*p* = 0.008) with ratings provided by the general public. Correlations were also observed with non-surgical physicians (*ρ* = −0.48, *p* = 0.03), non-plastic surgeons (*ρ* = −0.41, *p* = 0.07), and plastic surgeons (*ρ* = −0.32, *p* = 0.14). Negative correlation coefficients indicate that higher postoperative summary scores were associated with more favorable observer-based aesthetic ratings (lower scores).

**Table 5:** Correlation between the postoperative study-specific summary score derived from the selected FACE-Q scales and observer-based aesthetic evaluations.

Evaluator group	Spearman *ρ*	*p*-value
Plastic surgeons	−0.32	0.14
Non-plastic surgeons	−0.41	0.07
Non-surgical physicians	−0.48	0.03*
General public	−0.56	0.008*

For this exploratory analysis, a study-specific summary score was calculated for each patient as the arithmetic mean of the postoperative raw summed Face Overall, Natural Appearance, and Psychological Function scores. This derived measure was used solely for the present exploratory correlation analysis and does not represent a validated FACE-Q scale, domain score, composite score, or overall FACE-Q score. Median postoperative-only aesthetic scores were calculated for each patient within each evaluator group. Spearman's rank correlation coefficients were then calculated between the study-specific summary score and observer-based aesthetic ratings. Negative correlation coefficients indicate that higher summary scores were associated with more favorable observer ratings (lower aesthetic scores). Values marked with an asterisk indicate statistical significance (*p* < 0.05).

Numerically, the largest correlation coefficient was observed for the general-public ratings. However, because formal statistical comparisons between the correlation coefficients were not performed, these findings should be considered exploratory and should not be interpreted as demonstrating a statistically greater association with any particular evaluator group.

## 4 Discussion

The present study provides a structured, perception-based evaluation of facial local flap reconstruction by integrating paired ordinal analysis, contextual comparison, and evaluator stratification, thereby offering a comprehensive understanding of how aesthetic outcomes are interpreted across facial regions and professional backgrounds. Our findings demonstrate that exposure to the preoperative appearance of the lesion is associated with a systematic recalibration of aesthetic judgment, with postoperative outcomes consistently rated more critically after contextual comparison. A strength of this study is the inclusion of a relatively large and professionally diverse evaluator cohort. By incorporating 203 independent assessors from multiple professional backgrounds, this design improves the precision and breadth of the observed perceptual estimates compared with studies relying on smaller or more homogeneous observer groups. However, the size of the evaluator panel should not be interpreted as establishing external validity, as generalizability also depends on the recruitment strategy and the representativeness of the evaluators with respect to their respective target populations.

Across all facial regions analyzed, evaluators assigned significantly higher aesthetic scores following exposure to preoperative photographs, despite favorable postoperative-only ratings. This observation indicates that aesthetic assessment is not an isolated appraisal of reconstructed appearance but rather a cognitive process influenced by knowledge of the preoperative lesion and perceived reconstructive limitations. Similar perceptual shifts have been described in studies examining scar perception, facial asymmetry, and outcome reporting in reconstructive surgery, suggesting that aesthetic judgment evolves when reference points are altered (11–14).

Our findings also demonstrate that awareness of the preoperative lesion modifies how postoperative results are interpreted. Once observers were shown the appearance and extent of the preoperative lesion outcomes were viewed within a different evaluative framework. Although this contextual information influenced the interpretation of reconstructive results, perceptual differences among evaluator groups remained evident, indicating that professional background continues to shape aesthetic interpretation even when the preoperative condition is known. Importantly, this pattern persisted after accounting for the cross-classified dependency of ratings at both the patient and evaluator levels. The significant condition-by-evaluator category interaction suggests that the magnitude of contextual reassessment varied according to evaluator background, whereas the absence of a significant condition-by-region interaction supports a more cautious interpretation of apparent regional differences.

Importantly, postoperative-only median scores in our cohort corresponded to outcomes perceived as close to normal anatomy, supporting the effectiveness of region-specific local flap reconstruction. The increase in scores after contextual comparison should not be interpreted as diminished reconstructive success, but rather as evidence that evaluators apply stricter criteria once the extent of the preoperative lesion becomes apparent.

Descriptive region-specific analyses suggested variation in the magnitude of perceptual change across facial subunits, with numerically greater changes observed in the periocular and perioral regions. However, the cross-classified mixed-effects analysis did not demonstrate a statistically significant condition-by-region interaction. Accordingly, these apparent regional differences should be interpreted as exploratory rather than as evidence of an established hierarchy of contextual sensitivity. These findings align with the aesthetic subunit principle, which emphasizes that regions with high functional and visual prominence—such as the eyelids and perioral unit—are particularly sensitive to contour irregularities, scar visibility, and asymmetry. Even minor deviations in these areas may therefore attract greater aesthetic scrutiny.

Although malar reconstructions may theoretically benefit from greater tissue redundancy, smoother contour transitions, and improved scar camouflage, the present study included only two patients with malar defects. Therefore, the numerically smaller and more uniform perceptual changes observed in these cases should not be interpreted as evidence of intrinsically lower perceptual sensitivity of the malar region. Rather, these findings should be considered exploratory and hypothesis-generating until confirmed in larger and more balanced region-specific cohorts.

Stratification by evaluator background further demonstrated that professional expertise significantly influences aesthetic perception. Although all evaluator groups exhibited significant paired changes after contextual comparison, plastic surgeons consistently showed smaller perceptual shifts than non-plastic surgeons, non-surgical physicians, and members of the general public. This likely reflects greater familiarity with reconstructive constraints, scar maturation, and the trade-offs inherent in facial reconstruction. However, the interobserver agreement analysis demonstrated substantial heterogeneity within each evaluator category, with generally low ordinal Krippendorff's alpha values and particularly limited agreement during contextual assessment. Thus, although evaluator background was associated with the magnitude of contextual reassessment at the group level, individual aesthetic judgments remained heterogeneous within each professional category.

Conversely, evaluators without specialized reconstructive training demonstrated larger contextual shifts, particularly in the perioral and periocular regions. In the unadjusted region-specific analyses, inter-group differences reached statistical significance in the perioral and periocular regions. However, these findings should be interpreted in conjunction with the mixed-effects analysis and the limited number of independent patients within individual facial regions. Similar discrepancies between expert and non-expert observers have been reported in prior perception-based studies of facial reconstruction and cosmetic outcomes (7, 12, 15, 16).

Beyond defect closure and functional restoration, reconstructive procedures inherently involve an aesthetic dimension that becomes particularly central in facial reconstruction (6). Because the face plays a fundamental role in interpersonal communication and social identity, postoperative appearance is evaluated not only within the medical community but also within everyday social environments. Consequently, the perceptions of individuals without medical training may represent a particularly relevant reference point when assessing aesthetic outcomes. Evaluations restricted solely to surgeon-based perspectives may therefore provide an incomplete representation of reconstructive success. Understanding how reconstructed faces are perceived by members of the general public offers important insight into how surgical outcomes are interpreted in real-world social interaction (15, 17).

This difference in perception highlights an important aspect of reconstructive surgery. Surgeons often evaluate outcomes through the lens of technical feasibility, reconstructive limitations, and surgical achievement, whereas non-specialist observers interpret facial appearance without awareness of the underlying reconstructive complexity (18). Consequently, procedures regarded as highly successful within reconstructive surgery may still be perceived as residual deformities by individuals unfamiliar with the reconstructive context. Recognizing this perceptual gap may help improve communication with patients and facilitate more realistic discussions regarding expected postoperative appearance.

From a clinical perspective, these findings have several implications. First, they support the continued use of region-specific local flaps as reliable reconstructive options capable of achieving favorable aesthetic outcomes when assessed independently. Second, they highlight the value of contextual photographic comparison in both outcome evaluation and patient counseling, particularly for reconstructions involving visually sensitive facial subunits.

An additional dimension of this study was the incorporation of patient-reported outcomes using the FACE-Q. Improvements in Face Overall and Natural Appearance suggest that facial local flap reconstruction may provide meaningful subjective benefits beyond technical defect closure, whereas the absence of a significant change in Psychological Function indicates that broader psychosocial recovery may not necessarily parallel improvements in perceived facial appearance. Exploratory correlations between the study-specific summary score derived from the selected FACE-Q scales and observer-based ratings further suggest that patient-reported and externally perceived aesthetic outcomes may capture related, but not identical, dimensions of reconstructive success. Although the association appeared more pronounced for ratings provided by the general public, the correlation coefficients were not formally compared across evaluator groups; therefore, this observation should not be interpreted as evidence of greater agreement with any particular group. These findings support a multidimensional approach to outcome assessment that integrates patient-reported experience with observer-based aesthetic evaluation (19).

While the present study was intentionally designed to examine observer-dependent aesthetic perception and patient-reported outcomes, these measures capture a different dimension of reconstructive success from objective morphometric assessment. Three-dimensional stereophotogrammetry may provide a complementary approach by enabling quantitative assessment of postoperative facial morphology through landmark- and surface-based measurements. Caretto et al. recently demonstrated the feasibility of three-dimensional photogrammetry for objectively quantifying morphologic changes following oncologic nasal reconstruction and relating these measurements to subjective aesthetic ratings. Integration of objective three-dimensional morphometric analysis with observer-based perception and patient-reported outcomes may therefore provide a more comprehensive framework for evaluating facial reconstructive outcomes in future studies (20).

This study has several limitations. First, the number of patients within individual facial regions was limited, particularly in the malar subgroup, which included only two cases. Although each case was evaluated by a large number of observers, the small number of independent patients limits the generalizability of region-specific findings; therefore, comparisons among facial subunits should be regarded as exploratory. Second, aesthetic assessment was based on photographic evaluation rather than in-person examination. Because image acquisition was retrospective and was not performed according to a prospectively predefined photographic protocol, complete standardization of background, lighting, framing, camera distance, head position, and facial expression could not be ensured. Although photographs were selected and presented using a consistent evaluation format, these acquisition-related variations may have influenced observer-based aesthetic ratings and should be considered when interpreting the perceptual findings. Third, the sequential design of the photographic assessment represents an additional limitation. Because all evaluators first assessed the postoperative photographs alone and subsequently reassessed the same outcomes after viewing the corresponding preoperative photographs, the observed perceptual changes cannot be attributed exclusively to the introduction of preoperative context. Memory of the initial rating, anchoring effects, awareness of the sequential assessment process, or a tendency to reconsider a previous response may also have contributed to the observed changes. Future studies should consider randomized or counterbalanced presentation sequences, independent evaluator groups, or an appropriate washout period between assessments to better isolate the specific effect of preoperative contextual information. Although evaluator age and sex were recorded, these variables were not included as analytical factors in the original study design. Their potential influence on aesthetic judgments therefore cannot be excluded and should be considered when interpreting the findings. More detailed individual-level variables, including prior experience with facial reconstruction or oncologic surgery and detailed educational or occupational characteristics of the general-public group, were also not systematically recorded and may have contributed to heterogeneity within the evaluator groups. Furthermore, because evaluators were recruited by direct invitation rather than through population-based sampling, the evaluator cohort should not be considered fully representative of the broader professional or general-public populations. Finally, future studies with larger and more balanced region-specific cohorts and flap-type–specific analyses may provide a more comprehensive understanding of the factors influencing perceived aesthetic outcomes after facial reconstruction.

## 5 Conclusion

Aesthetic success after facial local flap reconstruction extends beyond technical surgical outcomes and is shaped by preoperative context and observer perspective. Although local flaps provided favorable postoperative outcomes, differences between specialist and non-specialist perceptions highlight the value of incorporating multiple perspectives into outcome assessment. Exploratory correlation analyses demonstrated associations of varying magnitude between the postoperative study-specific summary score derived from the selected FACE-Q scales and observer-based ratings; however, the correlation coefficients were not formally compared across evaluator groups. Integrating patient-reported outcomes with observer-based assessment may therefore provide a more comprehensive and patient-centered framework for evaluating facial reconstruction after cutaneous tumor resection.

## Data Availability

The original contributions presented in the study are included in the article/Supplementary Material, further inquiries can be directed to the corresponding author.
